# Tobacco: science, policy and public health

**DOI:** 10.1038/sj.bjc.6602440

**Published:** 2005-04-05

**Authors:** F A Stillman, H Wipfli

**Affiliations:** 1 Institute for Global Tobacco Control, Johns Hopkins Bloomberg School of Public Health, Baltimore, MD, USA

Tobacco: Science, Policy and Public health is a major reference text that is intended for an international public health audience, including epidemiologists, health economists and those working or interested in global tobacco control. The volume is primarily designed as a comprehensive review of the scientific literature of the health effects of tobacco use. This in itself is an enormous endeavor. This volume also provides information on the policy-relevant approaches undertaken to curb the use of tobacco and the public health lessons learned from the programmatic efforts to end the use of this deadly product. This is an extremely important topic as there are approximately 1.1 billion smokers around the world and the epidemic of tobacco-related disease is increasing. Based on current trends, by 2030 ten million people per year will die from tobacco-related deaths, accounting for one in every six deaths worldwide.

Despite the large number of chapter authors, the book flows reasonably well. The writing is clear and concise, making this an excellent resource for researchers as well as practitioners. In addition, the individual chapters are extremely well referenced, providing an appropriate starting place for health science students who wish to learn more about tobacco science and public health. A significant portion of this book contains a detailed synthesis of the literature related to active and passive smoking and health, including chapters on cancer, heart disease, respiratory disease, and other diseases. The book's editors have done a remarkable job of recruiting scientific leaders who have been on the cutting edge of tobacco control research since the 1950s to provide their unique perspectives. Sir Richard Doll, for example, provides rare historical insights into how the evidence linking tobacco and disease evolved during the past 50 years. Other chapters succinctly document the underlying biologic mechanisms that are related to addiction and disease and a detailed description of how cigarettes have been purposely engineered to be a disastrous nicotine delivery device since ‘carcinogens accompany nicotine in every puff’.

The book also provides a general history of how the epidemic developed and spread throughout the world. Case studies describing the tobacco epidemic in key countries, including Great Britain, India, and China, add specific details and depth to the global snapshot. Evidence-based tobacco control policies and other legal and advocacy strategies that have been utilised by the public health community to control tobacco promotion and use are also reviewed.

As we have indicated, this book does an excellent job in documenting the remarkable history of tobacco control research and the extensive evidence base that has been generated. However, while this evidence is essential, it is not sufficient in and of itself to generate sound public policy. Moreover, much of the evidence has been reviewed previously by the US Surgeon General and other scientific institutions. Unfortunately, despite all the evidence and understanding that has accrued about tobacco and health and the knowledge that has been gained to combat tobacco use around the world, national and international policy responses often fail to reflect this knowledge base. That is why it would have been beneficial for the editors to include a more comprehensive review concerning the development and implementation of strong and effective programmes and policies. The sections on the public health and policy approaches taken to change behaviour of individuals and of populations are given much less coverage when compared with that given to the global health burden. Descriptions of smoking patterns in selected countries are provided, but more information on the historical perspective of national and international tobacco control movements would have been beneficial. As tobacco control evolves in the coming years, it will be increasingly important to understand not just the health effects, the health burden and underlying genetic processes, but the political, social, cultural and economic factors that facilitate or inhibit public health policy.

‘If public health is to be more successful in the 21st century, it must comprehend the magnitude of the forces against it and the strategies used to engineer its defeat’ (McKinlay and Marceau, 2000). Nowhere is this more apparent than the field of tobacco use, as a leading cause of preventable death surrounded by a complex web of factors. While it is always important to discuss the negative health consequences caused by smoking, future texts on tobacco should also include a more comprehensive review of the best practices, policies and actions that need to be taken to actually reduce tobacco use in countries around the world.
